# Phylogenetic distribution, biogeography and the effects of land management upon bacterial non-specific Acid phosphatase Gene diversity and abundance

**DOI:** 10.1007/s11104-017-3301-2

**Published:** 2017-06-12

**Authors:** Andrew L. Neal, Martin Blackwell, Elsy Akkari, Cervin Guyomar, Ian Clark, Penny R. Hirsch

**Affiliations:** 1 Department of Sustainable Agricultural Sciences, Rothamsted Research, Harpenden, Hertfordshire, AL5 2JQ UK; 2 Department of Sustainable Agricultural Sciences, Rothamsted Research, North Wyke, Okehampton, Devon EX20 2SB UK; 3 Inria/IRISA GenScale, Campus de Beaulieu, cedex, 35042 Rennes, France

**Keywords:** Phosphorus, Phosphate, Acid phosphatase, Soil, Metagenomics, Exoenzymes

## Abstract

**Background and aims:**

Bacterial Non-Specific Acid Phosphatase (NSAP) enzymes are capable of dephosphorylating diverse organic phosphoesters but are rarely studied: their distribution in natural and managed environments is poorly understood. The aim of this study was to generate new insight into the environmental distribution of NSAPs and establish their potential global relevance to cycling of organic phosphorus.

**Methods:**

We employed bioinformatic tools to determine NSAP diversity and subcellular localization in microbial genomes; used the corresponding NSAP gene sequences to census metagenomes from diverse ecosystems; studied the effect of long-term land management upon NSAP diversity and abundance.

**Results:**

Periplasmic class B NSAPs are poorly represented in marine and terrestrial environments, reflecting their association with enteric and pathogenic bacteria. Periplasmic class A and outer membrane-associated class C NSAPs are cosmopolitan. NSAPs are more abundant in marine than terrestrial ecosystems and class C more abundant than class A genes, except in an acidic peat where class A genes dominate. A clear effect of land management upon gene abundance was identified.

**Conclusions:**

NSAP genes are cosmopolitan. Class C genes are more widely distributed: their association with the outer-membrane of cells gives them a clear role in the cycling of organic phosphorus, particularly in soils.

## **Introduction**

The biogeochemical cycling of phosphorus (P) is important to biological productivity on a global scale: fixation of both carbon dioxide and nitrogen in the oceans by the cyanobacterium *Trichodesmium* is limited by the availability of inorganic P (Moore et al. 2013) and global agricultural production relies upon regular inorganic P inputs to soil (derived primarily from finite rock phosphate reserves) to maintain yields (Cordell et al. 2009). Bacteria commonly express hydrolase enzymes to acquire nutrients from complex organic molecules: for example, cellulases to acquire carbon from cellulose or chitinases to acquire nitrogen from chitin. There are several described enzyme families that can dephosphorylate organic compounds to acquire P, among them phosphatases and phytases. Many of these hydrolase enzymes may be secreted outside the cell, where they either function as soluble enzymes or are retained as membrane-bound enzymes to hydrolyse organic compounds into inorganic phosphate and organic by-products that can be transported across membranes.

Among phosphoric monoester hydrolases, alkaline phosphatases (EC 3.1.3.1) are the most commonly studied. There are three gene families, *phoA*, *phoD* and *phoX* which code for enzymes with monoester and some diester activity. Similarly, there are a number of phytase (EC 3.1.3.8 and EC 3.1.3.26) families, differentiated by different catalytic mechanisms and co-factor requirements (Mullaney and Ullah 2003). Alkaline phosphatase and phytase genes are distributed across a broad phylogenetic range and display a high degree of microdiversity (Lim et al. 2007; Zimmerman et al. 2013; Ragot et al. 2015) where ecologically- or physiologically-distinct groups exist within phylogenetically-related clades. In marine systems, there is evidence that alkaline phosphatase genes *phoD* and *phoX* are more abundant than *phoA* (Luo et al. 2009; Sebastian and Ammerman 2009) and the β-propeller phytase gene is the most abundant phytase (Lim et al. 2007). Additionally, *phoD* is the dominant alkaline phosphatase gene in terrestrial ecosystems (Tan et al. 2013) and more abundant in soils than other environments (Ragot et al. 2015). From a functional standpoint, abundance of *phoD*-like sequences, assessed by quantitative PCR, correlate well with estimates of potential alkaline phosphatase activity (Fraser et al. 2015) in soils, but there is little information regarding other alkaline phosphatases or phytases. These genes collectively appear to be regulated by the availability of P via by the Pho regulon (Vershinina and Znamenskaya 2002) and are only expressed under conditions of P-limitation.

There is a third group of phosphoric monoester hydrolases which exhibit optimal in vitro activity at low pH and are therefore termed acid phosphatases (EC 3.1.3.2). Based upon amino acid sequence analysis, these enzymes are separated into three distinct types (referred to as classes A, B and C) but all lack strong substrate specificity, instead showing activity across a broad range of structurally-unrelated phosphoesters (Rossolini et al. 1998): in recognition of this, the enzymes are termed collectively non-specific acid phosphatases (NSAPs). NSAPs of bacteria and archaea share a common evolutionary origin (Gandhi and Chandra 2012) and appear to be distributed widely among prokaryotes and eukaryotes. At least one NSAP gene, class A *phoC* of the bacterium *Morganella morganii*, appears not to be regulated by the availability of P (Thaller et al. 1994) and thus may be regulated in a different manner from alkaline phosphatases and phytases. The significance of NSAPs with respect to P-acquisition in soils and other environments is rarely considered and poorly understood. However, because of their different physiological response traits (pH, specificity etc.) and potentially different regulation compared to alkaline phosphatases, environmental information regarding the biogeographical distribution of NSAPs and the effects of land management upon their abundance is critical to development of mechanistic models of dynamic biological processes regarding P cycling in the environment. The work described here begins to address this lack of knowledge by first surveying the distribution of NSAP classes in sequenced microorganisms then, using the assembled sets of class A, B, and C NSAP gene sequences, assessing the distribution and abundance of NSAPs in publically-available metagenome sequences from geographically- and physically-diverse environments and finally, studying the effects of land management on NSAP abundance in a well-studied long-term field experiment.

## **Materials and methods**

### **Distribution of NSAP classes in sequenced microorganisms**

Archetypal proteins for each NSAP class listed by Gandhi and Chandra (2012) were used as starting points to generate sets of reference proteins for each NSAP class. JackHMMER, part of the HMMER ver. 3.1b1 software suite, was used to generate a collection of homologous protein sequences for each group of archetype protein sequences from the UniprotKB database, as well as generating a profile hidden Markov model (pHMM) for each enzyme (Durbin et al. 1998; Eddy 2011). Cut-off Expect (*E*) values were adjusted for each protein family, however values were typically 1 × 10^−80^ ≥ *E* ≥ 1 × 10^−100^. The BLOSUM45 substitution scoring matrix was used, together with gap opening and gap extension penalties of 0.02 and 0.4 respectively. For each JackHMMER iteration, only sequences having identical domain architecture as the query sequence were used to generate the pHMM for the proceeding iteration. Iterations were continued until no new sequences were included in the set. Each reference set of proteins was then edited manually to remove protein fragments. Any sequence with a length less than 70% that of the archetype was considered a fragment. Redundant protein sequences were removed using CD-HIT (Li and Godzik 2006). A multi-sequence alignment (MSA) of the remaining unique, full-length protein sequences was generated using the E-INS-i iterative refinement method using weighted-sum-of-pairs and consistency scores in MAFFT ver. 7.182 (Katoh and Standley 2013) employing BLOSUM62 and a gap opening penalty of 1.53. Maximum-Likelihood (ML) phylogenetic trees were generated using RAxML ver 7.2.8 (Stamatakis 2006), employing the PROTGAMMA amino acid substitution evolutionary model and Dayhoff matrix and bootstrapping, the number of bootstrap replicates determined using autoMRE convergence checking. Best-scoring ML trees were visualised using iTOL ver 3.2.4 (Letunic and Bork 2016).

### **NSAP protein localization**

To determine the subcellular localization of NSAP proteins we employed PSORTb ver. 3.0.2 (Yu et al., 2010). PSORTb employs support vector machines for each of the nine sub-cellular locations of Gram-negative and Gram-positive Eubacteria, and Archaeal prokaryotes, combining these predictions with results of BLASTP searches (*E* < 1 × 10^−9^) against reference sets of proteins of known subcellular localization and model HMMs for transmembrane-spanning α-helices and signal peptide cleavage sites. A Bayesian network is then used to combine predictions from all modules and generate a weighted localisation prediction based on the performance accuracies of each prediction module.

### **Metagenome analysis**

We studied a number of publically-available shotgun metagenomic datasets from diverse environments to determine the global distribution of the three NSAP classes (Table 1). Shotgun metagenome sequences generated using Illumina® sequencing technology containing at least 10 million reads were downloaded in FASTQ format from the European Nucleotide Archive (ENA), the DNA Data Bank of Japan and Sequence Read Archive. The specific datasets used were chosen because they contain the largest number of reads in each respective collection. We used three marine datasets, a bathypelagic sediment from the Gulf of Mexico (SRR4027974), sediment from the Columbia River estuary coastal margin, Washington State, US (ERR864075), and sediment from the Noosa River estuary in south east Queensland, Australia (ERR688352) and four terrestrial soil datasets, a tallgrass prairie soil from Fricke Cemetery, NE (ERR346662), a peat bog hydric soil from the Marcell Experimental forest, MN (SRR1157608), a rice paddy hydric soil from southeast China (SRR1190306), and an arid soil from Uluṟu, Northern Territory, Australia (ERR671923). We also included a managed grassland from the Highfield Ley-Arable long-term field experiment, Rothamsted, UK (experiment described below). Sequences were limited to a minimum quality score of 25 using a sliding window of 4 bases, and a minimum read length of 70 bases using Trimmomatic (Bolger et al. 2014)*.*

**Table 1:** Details of the publically-available metagenome sequence data sets compared in this study

Terrestrial Soil	Metagenome ID	Sequencing Technology	Number of Reads	Mean normalized counts of single-copy genes	Reference
Arid Soil, Uluṟu, Northern Territories Australia	ERR671923^a^	Illumina, HiSeq 2500	34,457,076	2982	www.bioplatforms.com/soil-biodiversity/
Rice Paddy Soil, South China	SRR1190306^b^	Illumina, HiSeq 2000	26,612,780	1707	
Tall grass prairie, Fricke Cemetery, Nebraska, USA	ERS351497^a^	Illumina, HiSeq 2000	41,112,030	2838	Fierer et al. 2013
Ombrotrophicbog soil, 75 cm subsurface, Marcell Experimental Forest, Minnesota, USA	SRR1157608^a^	Illumina, HiSeq 2000	51,628,818	3599	Lin et al. 2014
Marine Sediment					
Gulf of Mexico, bathypelagic (>1.5 km depth)	SRR4027974^c^	Illumina, HiSeq 2000	310,990,307	13,444	Mason et al. 2014
Columbia River costal margin, Washington, USA (33 ‰ salinity, 16 m depth)	ERR864075^a^	Illumina, HiSeq 1000	51,801,990	7528	Fortunato and Crump 2015
Noosa River estuary, Queensland, Australia (0.34 m depth)	ERR688352^a^	Illumina, HiSeq 2000	129,525,367	10,405	

^a^European Nucleotide Archive

^b^DNA Data Bank of Japan

^c^Sequence Read Archive

We adopted an assembly-free, gene-centric approach, MApPP (Metagenomics/transcriptomics Assignment pHMM Phylogenetic Placement), to analysing the abundance and phylogenetic diversity of NSAP genes associated with microbial communities in the different soils. For each collection of reference proteins, UniProtKB protein accessions were mapped to their respective ENA nucleotide sequence accession and the nucleic acid sequences were obtained. The resulting set of nucleotide sequences were aligned using the FFT-NS-i iterative algorithm in MAFFT, employing the 1PAM/κ = 2 scoring matrix and a gap opening penalty of 1.53. For each gene, a pHMM of the resulting MSA was generated using HMMbuild (HMMER ver 3.1b1) resulting in a 799 nucleotide (nt) Class A pHMM based upon 426 sequences, a 714 nt Class B pHMM based upon 319 sequences and an 836 nt Class C pHMM based upon 479 sequences. These pHMMs were used to search the unassembled metagenome datasets using HMMsearch, employing an *E* < 1 × 10^−5^ along the full sequence length. The probability thresholds for the multiple segment Viterbi, Viterbi and Forward filters were 0.02, 0.001 and 1 × 10^−5^ respectively.

PHMMER was used to compare the retrieved metagenome sequences, following six-frame translation using EMBOSS Transeq (Rice et al., 2000), to the UniprotKB protein sequence database to confirm that the sequences represented the correct protein family. Only those metagenome sequences for which one of the six frame translations elicited a UniprotKB hit of the appropriate protein family (*E* < 1 × 10^−5^) was included in the subsequent analysis. Phylogenetic placement and visualization of the recovered metagenome sequences upon reference Maximum-Likelihood phylogenetic trees was performed using pplacer ver 1.1 (Matsen et al., 2010).

To allow meaningful comparison between metagenomic datasets, gene abundance was expressed as a proportion of the estimated total number of genomes in each dataset, assessed by estimating the abundance of the ubiquitous, single-copy genes *rpoB*, *recA*, *gyrB* (Santos & Ochman, 2004) and *atpD* (Gaunt et al., 2001). Nucleotide sequence-based pHMMs were developed for each gene as described above. Metagenome-derived homologue counts for each single-copy gene were size-normalized to the length of the shortest gene, *recA* accounting for differences in length between the genes. To do this, the modal length of *recA* (1044 nt) was divided by the modal length of the other single-copy genes (1422 nt for *atpD*, 2415 nt for *gyrB*, 4029 nt for *rpoB*), and this value was then multiplied by each single-copy gene count. The size-normalized abundance of each target phosphatase gene was then calculated for each soil as [target gene count·read length/(mean normalized counts of single-copy genes)] (Howard et al., 2008). The mean normalized counts of single-copy genes for each metagenome is given in Table 1.

### Identification and phylogenetic placement of metagenome reads from Highfield soil treatments

#### **Soils**

Experimental soils were collected from permanent grassland, arable and bare fallow plots of the Highfield Ley-Arable experiment (00:21:48 °W, 51:48:18 °N) at Rothamsted Research. The soil is a silty clay loam (27% clay) (Chromic Luvisol according to FAO criteria). At the time of sampling, arable plots had been under continuous wheat rotation and receiving fertilization and pesticides according to normal farm management for 62 years, bare fallow plots had been maintained crop- and weed-free by regular tilling for 52 years, and grassland plots had been maintained as a managed sward of mixed grasses and forbs for over 200 years: all plots are considered now to be in quasi-equilibrium (Wu et al. 2012). Physical and biological data has already been reported for these soils (Table 2).

**Table 2:** Summary physical and chemical data of Highfield Ley-Arable experiment soils

	pH^a^ (H_2_O) / -log(g[H^+^]L^−1^)	Organic Carbon^a^ / mg g^−1^ soil	Free Organic Carbon^b^ / μg g^−1^ soil	Intra-aggregate Organic Carbon^b^ / μg g^−1^ soil	Nitrogen^a^ / μg g^−1^ soil	NaOH-EDTA extractable orthophosphate / μg g^−1^ soil	NaOH-extractable orthophosphate monoester / μg g^−1^ soil	NaOH-extractable pyrophosphate / μg g^−1^ soil
Arable	5.8	1.3	370	490	150	203	82	17
Bare Fallow	5.1	0.8	150	380	100	103	53	5
Grassland	6.0	3.9	4690	3010	390	240	139	10

^a^Gregory et al. 2016

^b^Hirsch et al. 2009

#### **Phosphorus chemistry in Highfield ley-arable experiment soils**

We employed alkaline ethylenediaminetetraacetic acid (EDTA) extraction (Bowman & Moir, 1993) to estimate the amounts of orthophosphate and organic P in each soil. 30 mL of a 250 mM NaOH:50 mM Na_2_-EDTA solution was used to extract P-compounds from 1.5 g of air dried, sieved (<2 mm) soil. Extraction was allowed to proceed for 14 h at 22 °C with continuous shaking. The resulting solution was centrifuged at a maximum relative centrifugal force of 13,416 (10,000 rpm, *r*_max_ 12 cm.) for 30 min and the supernatant collected. For solution ^31^P–NMR, 1 mL of a 50 μg-P L^−1^ methylenediphosphonic acid (MDPA, Sigma-Aldrich; M9508; ≥99%) solution was added to 20 mL of the remaining extractant as an internal standard. Following mixing, each sample was frozen and lyophilised in preparation for NMR analysis (Turner et al. 2003). Once dry, the sample was re-dissolved in 0.1 mL D_2_O and 0.9 mL of a 1 M NaOH, 0.1 M Na_2_-EDTA solution and transferred to a 5 mm NMR tube. Solution ^31^P–NMR spectra were collected on a DRX-500 spectrometer (Bruker UK Ltd.) operating at 202.456 MHz. A 6.2 μs pulse was used with a 0.41 s acquisition time and a delay of 2 s to collect spectra. Approximately 32,000 scans were acquired for each sample and broadband proton decoupling was applied (Cade-Menun and Liu 2014). Spectra were plotted with a line broadening of 2.5 Hz and chemical shifts of signals were determined in ppm relative to the orthophosphate peak which was set to 6 ppm. Peaks were identified by comparison with literature reports of shifts for model P compounds dissolved in NaOH-EDTA (Turner et al. 2003; Cade-Menun and Liu 2014). Processing of spectra was performed with ACD/1D NMR Processor and Manager Ver 12 (Advanced Chemistry Development, Inc., USA). Peak areas were calculated by integration and concentrations were calculated based upon the area of the MDPA peak at δ = 17.17 ± 0.01 ppm (*n* = 9). Inorganic orthophosphate at δ = 6 ppm, phosphate monoesters at δ = 3.11 to 5.58 ppm, pyrophosphate at δ = −4.03 to −4.07 ppm, diesters (DNA) at δ = 0.92 to 0.96 ppm, and phosphonates at δ = 16.15 ppm were the main groups identified.

#### **DNA extraction and Metagenome sequencing**

Soil was collected from triplicate plots for each treatment from the Highfield Ley-Arable experiment in October 2011 to a depth of 10 cm using a 3 cm diameter corer. The top 2 cm of soil containing root mats and other plant detritus was discarded. Ten cores per plot were pooled and thoroughly mixed whilst sieving through a 2 mm mesh; samples were then frozen at −80 °C. All implements were cleaned with 70% ethanol between sampling/sieving soil from each plot. Soil community DNA was extracted from a minimum of 2 g soil using the MoBio PowerSoil® DNA isolation kit (Mo Bio Laboratories, Inc. Carlsbad, CA) with three replicates for each soil treatment. When necessary, extracts were pooled to provide sufficient material for sequencing. 10 μg of high-quality DNA was provided for sequencing for each of the nine plots. Shotgun metagenomic sequencing of DNA from each soil was provided by Illumina® (Cambridge, UK) using a HiSeq™ 2000, generating 150 bp paired-end reads. Sequences were limited to a minimum quality score of 25 and a minimum read length of 70 bases using Trimmomatic (Bolger et al. 2014).

#### Statistical analysis

We used parametric one-factor Analysis of Variance tests for comparison of P concentrations and normalised abundance of NSAP classes in different environments and soil treatments. Where a significant treatment effect was determined (α = 0.05), means were compared post hoc employing the Holm-Šídák all pairwise multiple comparison procedure (SigmaPlot ver. 13, SysStat Software Inc.). The abundance of NSAP gene ecotypes was related to edaphic factors using canonical correspondence analysis (CCA) in PAST ver 3.15 (Hammer et al. 2001). Counts of individual ecotypes acted as “species” data: counts less than 5 were treated as 0 and ecotypes which were present in only 1 of the 9 soils were removed from the analyses. The pH, organic carbon (C_org_), Nitrogen (N) and NaOH-EDTA extractable orthophosphate concentrations in Table 2 were used to represent soil chemical environments and the ratio of intra-aggregate C_org_ to free C_org_ (intra-aggregate ratio) - calculated from data in Table 2 - was used to represent soil physical structure. C_org_ and N were significantly correlated in the soils (*r* = 0.999, *p* < 0.001), consequently only C_org_ was included in CCA. Variables were included in a single model combining both genes; significance was estimated based upon 9999 Monte Carlo permutations.

## Results

### Phylogenetic distribution and predicted subcellular localization of NSAP classes in sequenced bacterial genomes

#### **Class A**

**Fig. 1:**
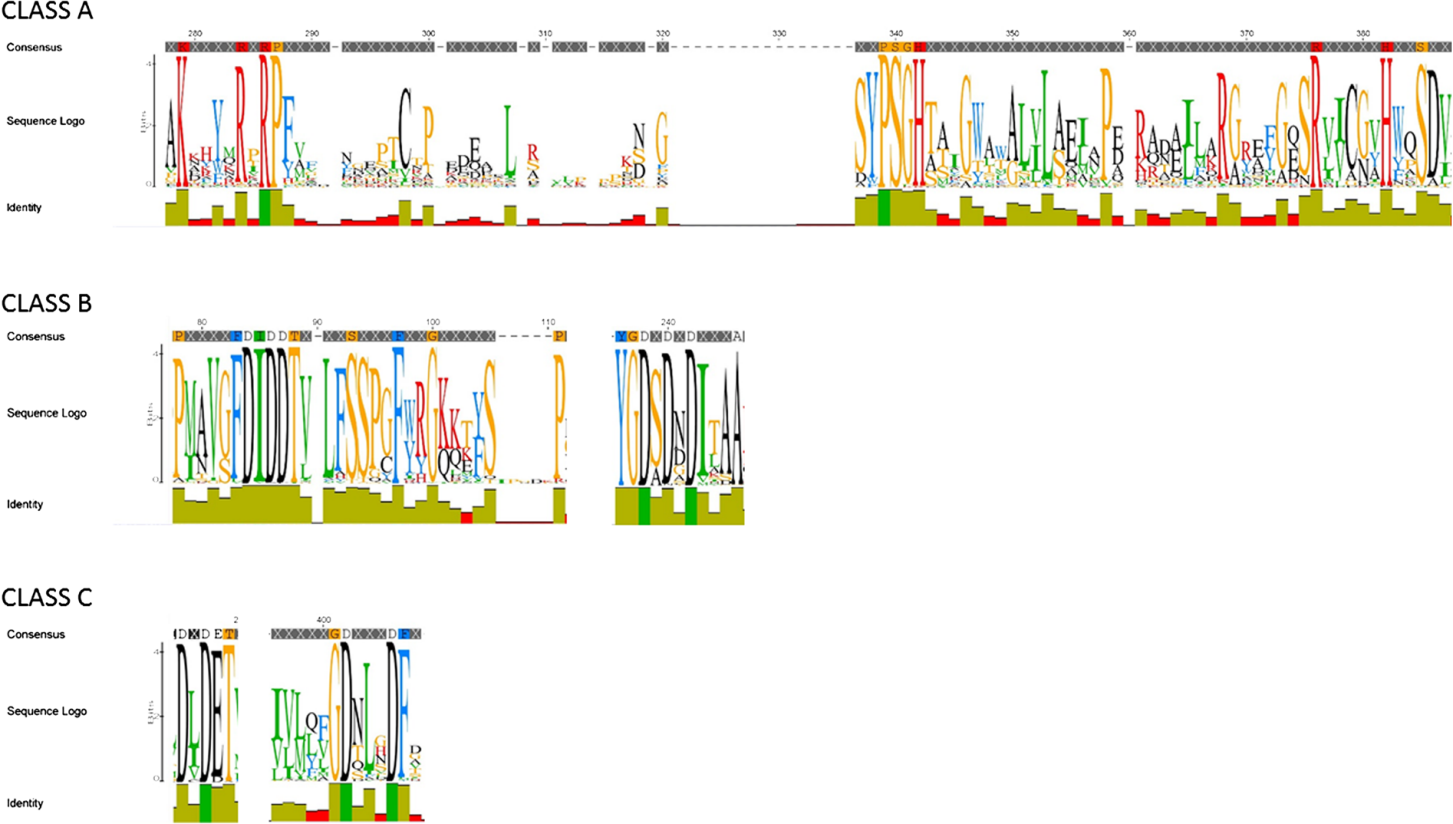
Sequence logos showing regions of conserved amino acid motifs for each class of non-specific acid phosphatase. Each logo was generated from multi-sequence alignments of collections of reference protein sequences and depicts the consensus sequence and diversity of the sequences

Using the pHMM-based JackHMMER routine, 376 unique class A NSAP proteins were identified in UniprotKB, all of them, apart from one uncultured microorganism, Gram-negative bacteria. The sequences shared the conserved amino acid motif KX_6_RP-(X_12–54_)-PSGH-(X_31–54_)-SRX_5_HX_2_D (Fig. 1) characteristic of class A NSAPs (Stukey and Carman 1997) and also shared with several lipid phosphatases and mammalian glucose-6-phosphatases. Predictions of subcellular localization of the proteins indicated that many were of indeterminate localization, however 61 of the proteins could be assigned a sub-cellular compartment. Of these, 27 were predicted to be periplasmic, 4 cytoplasmic and 30 were predicted with less certainty to be associated with the cytoplasmic membrane. Signal peptides were identified in all but 20 of the proteins, suggesting that most Class A proteins are transported at least out of the cytoplasm. However, it is clear that PSORTb was unable to determine a clear location for the proteins, possibly because NSAPs are a relatively poorly studied group and there may be few Class A NSAP proteins in the training datasets as a consequence. The proteins were found in a range of free-living bacteria including *Caulobacter*, *Stenotrophomonas*, *Methylobacterium*, *Sphingomonas*, *Xanthomonas* and *Pseudomonas*.

#### **Class B**

In all, 319 unique class B proteins were identified. The proteins were found predominantly in eukaryote-associating Gram-negative bacteria, for example *Aeromonas*, *Aggrigatibacter*, *Citrobacter*, *Enterobacter*, *Escherichia*, *Klebsiella*, *Photobacterium*, *Salmonella,* and *Xenorhabdus*. The protein sequences shared common conserved amino acid motifs PX_4_FDIDDTXVLFSSPXF at the N-terminal and YGD(S/A)DXDX_3_A at the C-terminal (Fig. 1) consistent with previous descriptions (Thaller et al. 1998). The majority of class B proteins tested (96%) were predicted to be periplasmic, the remaining 4% were of indeterminate location.

#### **Class C**

Of the three classes studied here, class C contained the greatest number of unique protein sequences, 1125, characterised as containing four highly conserved aspartic acid residues (D) (Fig. 1) across the two amino acid motifs DXDET at the N-terminal and GDX_3_DF at the C-terminal (Thaller et al. 1998). Approximately 90% of the proteins were predicted to be outer membrane-associated, less than 1% were predicted to be periplasmic and 9% were of indeterminate localisation. The proteins were identified in a broad range of bacteria including *Bacillus*, *Clostridium*, *Enterobacter*, *Erwinia*, *Lysobacter*, *Pedobacter*, *Pseudomonas*, *Rhodobacter* and *Serratia* among others.

#### **Biogeography of NSAPs**

**Fig. 2:**
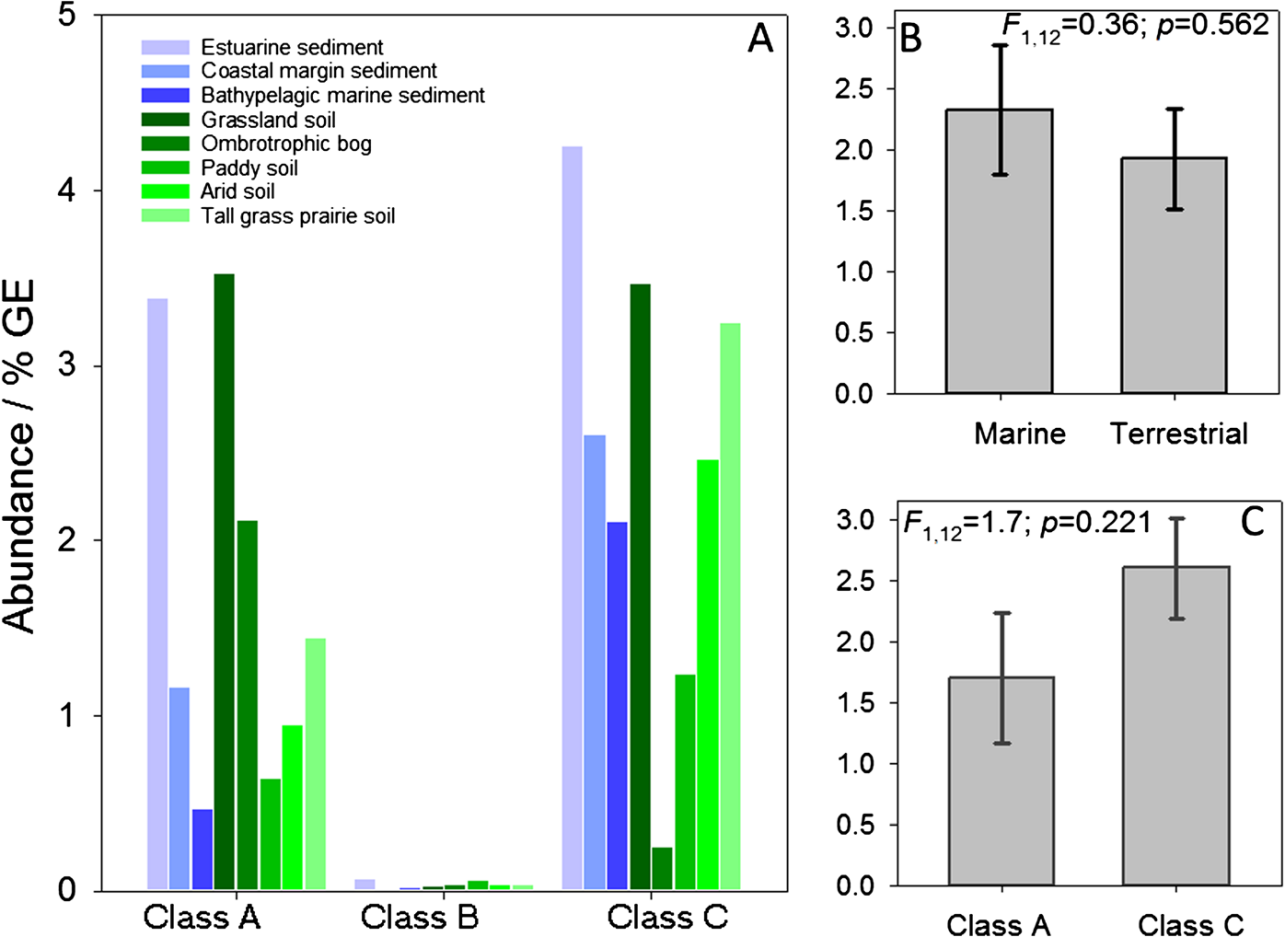
Relative abundance of non-specific acid phosphatase gene homologues in shotgun metagenomes developed from marine and terrestrial environments. The counts of homologous sequences are normalized relative to the number of genome-equivalents in each metagenome, normalization details are given in Materials and Methods section. **a** – Relative abundance of Class A*,* B and C NSAPs in each environment. **b** – Mean relative abundance of class A and B NSAPs in marine and terrestrial environments. **c** – Mean relative abundance of class A and B NSAPs in the datasets overall

Shotgun metagenome datasets from eight diverse marine and terrestrial environments were used to establish the distribution of NSAPs. Gene abundance was normalized and expressed as % genome equivalents (%GE) to allow meaningful comparison between metagenome datasets of different read numbers. A census of the environments (Fig. 2) indicated that classes A and C are relatively much more abundant in both marine and terrestrial environments than class B. Given the association of class B with pathogens and other microorganisms found in close association with eukaryotes this is perhaps unsurprising. Comparisons of the normalized abundance of classes A and C indicated that although both are relatively more abundant in marine than terrestrial environments, there was no significant difference in abundance (*F*_1,12_ = 0.36; *p* = 0.526). Similarly, although class C NSAPs were more abundant across the eight environments than class A, again differences were not significant (*F*_1,12_ = 1.7; *p* = 0.221). Comparison of the normalized abundance of classes A and C (< 5%GE) with the abundance of the alkaline phosphatase *phoX* in marine systems (relative to *recA*, Sebastian and Ammerman 2009) indicates that NSAPs are considerably less common than *phoX* (18%GE).

**Fig. 3:**
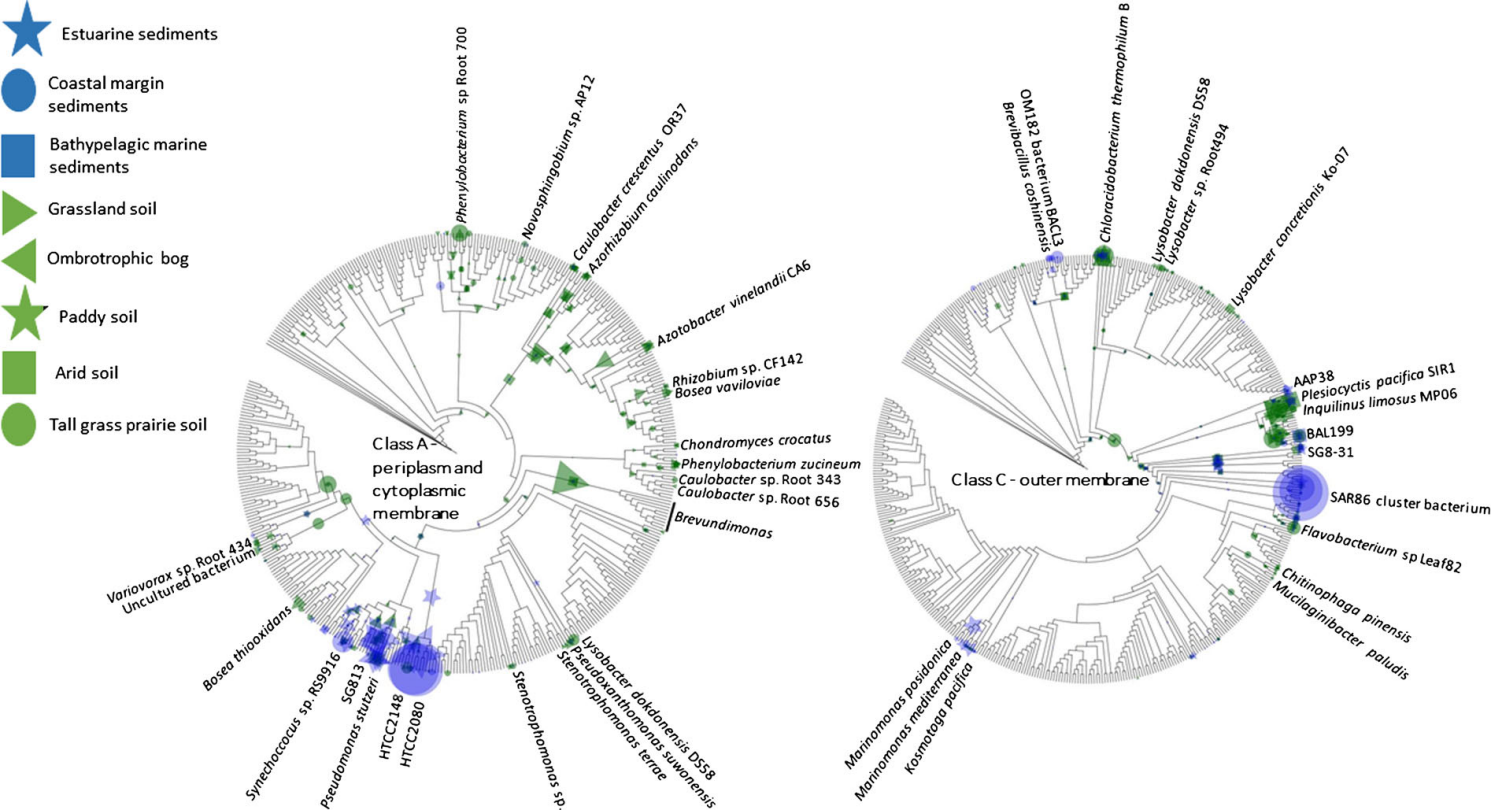
Phylogenetic placement of metagenome sequences generated from contrasting marine and terrestrial environments showing homology to class A and class B non-specific acid phosphatase reference gene sequences. The placement of reads from each of the eight metagenomes are overlaid for each maximum-likelihood tree and are represented by different symbols. In each case, the normalized abundance of each ecotype (accumulation of reads) is represented, normalized to the maximum abundance - HTCC2148 in the coastal margin sediment sample in the case of Class A and SAR86 cluster bacterium, again in the coastal margin sediment in the case of Class C. The size of the symbol representing read placements is proportional to the normalized relative abundance. For clarity, organisms harbouring homologous gene sequences are only identified where they are associated with read placements. Geographical location of the different soils and sediments is given in Table 1

To compare the phylogenetic distributions, the confirmed homologous metagenome sequences were placed on respective class A and class C ML phylogenetic trees. The normalized abundance (%GE) of the read placements is shown on the phylogenetic trees. In the case of class A genes, there was a clear association between gene phylogeny and environment (Fig. 3). For example, the dominant class A gene in the Columbia River estuary shows high homology to that of marine *Gammaproteobacteria* HTCC2080 and HTCC2148, originally isolated from nearby coastal waters off Oregon (Thrash et al. 2010). A second placement, with high homology to the cyanobacterium *Synechoccocus* sp. RS9916, is also abundant in the Columbia estuary. In contrast, sequences from the Noosa River estuary generally have weaker homology to the sequence database and the class A sequences appear more diverse than those from the Columbia estuary. Sequences with reduced homology to HTCC2080 and HTCC2148 are most abundant, but a group of sequences with varying homology to class A NSAPs of *Methylococcus capsulatus* and *Pseudomonas stuzeri*, the *Deltaproteobacterium* SG813 and a second group with varying homology to *Synechococcus* spp. are also present as well as sequences with high homology to *Pseudohongiella spirulinae*. The least diverse and least abundant of the three marine environments was the bathypelagic sediment. The limited number of sequences from these sediments were most closely related to isolate SG813 and *Desulfotalea psychrophila*. In contrast to marine environments, NSAP class A compliments from soils were relatively diverse: the phylogenetic distribution of genes was more diverse and the dominant ecotype differed between environments. For example, NSAP class A was most numerous in terrestrial environments in the peat bog of Marcell Experimental Forest. Here the greatest number of metagenome sequences exhibited homology to *Methylobacterium* spp. and *Granulibacter* spp. Some Marcell placements showed very similar homology to *M. capsulatus* and *P. stuzeri* as those from the Columbia estuary. Although less abundant, these placements were also associated with the Paddy soil metagenome. These placements were relatively less abundant in either the Fricke prairie or Uluṟu grassland metagenomes which were dominated by reads with homology to *Phenylobacterium* sp., *Lysobacter dokdonensis* DS58, *Azotobacter vinelandii* CA6, reads with low homology to a group of *Alphaproteobacteria* and a number of read placements with limited homology to various *Proteobacteria* including *Brevundimonas* spp.

For class C NSAPs there was again a distinct separation in marine and terrestrial environments in the placement of metagenome reads on the reference ML phylogenetic tree (Fig. 3). The Columbia estuary was dominated by a group of closely-related reads with high homology to NSAP from a SAR86 cluster bacterium and lesser numbers of reads with high homology to class C NSAPs from OM182 (oligotrophic marine) bacterium BACL3, *Brevibacillus choshinensis* and *Chloracidobacterium thermophilum* B. Again, the population of class C NSAPs in the Noosa estuary were distinct from the Columbia estuary, showing homology to *Marinomonas* spp. and *Kosmotoga pacifica*, *Plesiocystis pacifica* SIR1, *Alphaproteobacterium* AAP38, *Gammaproteobacterium* SG8–31, and a *Chlorobium* spp. clade. The Gulf of Mexico again showed the least number of class C reads of the marine environments and was dominated by reads with high homology to the class C NSAP of *Alphaproteobacterium* BAL199, a *Rhodopirellula* spp. clade and the same *Chlorobium* spp. clade identified in Columbia River. A smaller numbers of reads showed high homology to *Vibrio caribbeanicus* and *B. chishinensis*. For terrestrial soils, the Marcell peat bog contained few class C NSAPs and what metagenome reads were identified typically showed limited homology to reference genes. In contrast, the datasets from paddy soil, Fricke prairie and Uluṟu contained greater numbers of class C homologous reads: those identified exhibited greater homology to reference genes. Reads from paddy soil displayed homology to a group of *Alphaproteobacteria* including *Azospirillum* and *Hyphomonas* and there were also placements of small numbers of reads distributed across the reference tree. The metagenomes from Fricke prairie and Uluṟu shared a number of common placements: in both environments reads with high homology to *Azospirillum* spp. were identified as well as reads with varying homology to a number of other *Alphaproteobacteria* including bacterium BAL199 (also identified in the Gulf of Mexico), *Martelella endophytica*, *Thalassospira* spp., *C. thermophilum* B (also identified in the Columbia River estuary) and *Lysobacter* spp. Homologous sequences of the *Delataproteobacterium P. pacifica* SIR1 were also present in both environments, as well as the Noosa River estuary and Gulf of Mexico.

#### **Effect of land management upon abundance and diversity of NSAPs in soil**

To evaluate the potential effect of land management upon abundance and diversity of NSAP genes in soil we studied the Highfield Ley-Arable Experiment. A great deal of physical and biological data has already been reported for these soils (Hirsch et al. 2009; Wu et al. 2012; Gregory et al. 2016; Hirsch et al. 2016). Quantification of orthophosphate, orthophosphate monoester and pyrophosphate moieties in soil extracts by ^31^P–NMR indicated significant differences between the three soil managements in the amount of total P, summed from NMR spectra of NaOH-EDTA extracts (*F*_2,18_ = 57.0; *p* < 0.001: all soil managements were significantly different from each of the others, smallest difference = 29.0 μg g^−1^, *t* = 4.02, *p* < 0.001). Comparison of the amounts of different moieties in the three soils indicated that the mean concentration of orthophosphate (239.7 μg g^−1^ ± 18.5 μg g^−1^ SE, 203.1 ± 10.9 μg g^−1^, 102.5 ± 7.4 μg g^−1^ for grass, arable and bare fallow soil respectively) and orthophosphate monoesters (138.6 μg g^−1^ ± 2.1 μg g^−1^ SE, 81.5 ± 6.6 μg g^−1^, 52.5 ± 1.4 μg g^−1^ for grass, arable and bare fallow soil respectively) were significantly different in each of the soils (smallest difference = 28.9 μg g^−1^, orthophosphate monoesters in arable versus bare fallow soil: *t* = 2.3, *p* = 0.032). There was no significant difference in mean concentrations of pyrophosphate between the soils (9.9 μg g^−1^ ± 1.2 μg g^−1^ SE, 16.7 ± 11.6 μg g^−1^, 4.5 ± 0.4 μg g^−1^ for grass, arable and bare fallow soil respectively; largest difference = 12.2 μg g^−1^, arable versus bare fallow soil: *t* = 0.98, *p* = 0.716).

**Fig. 4:**
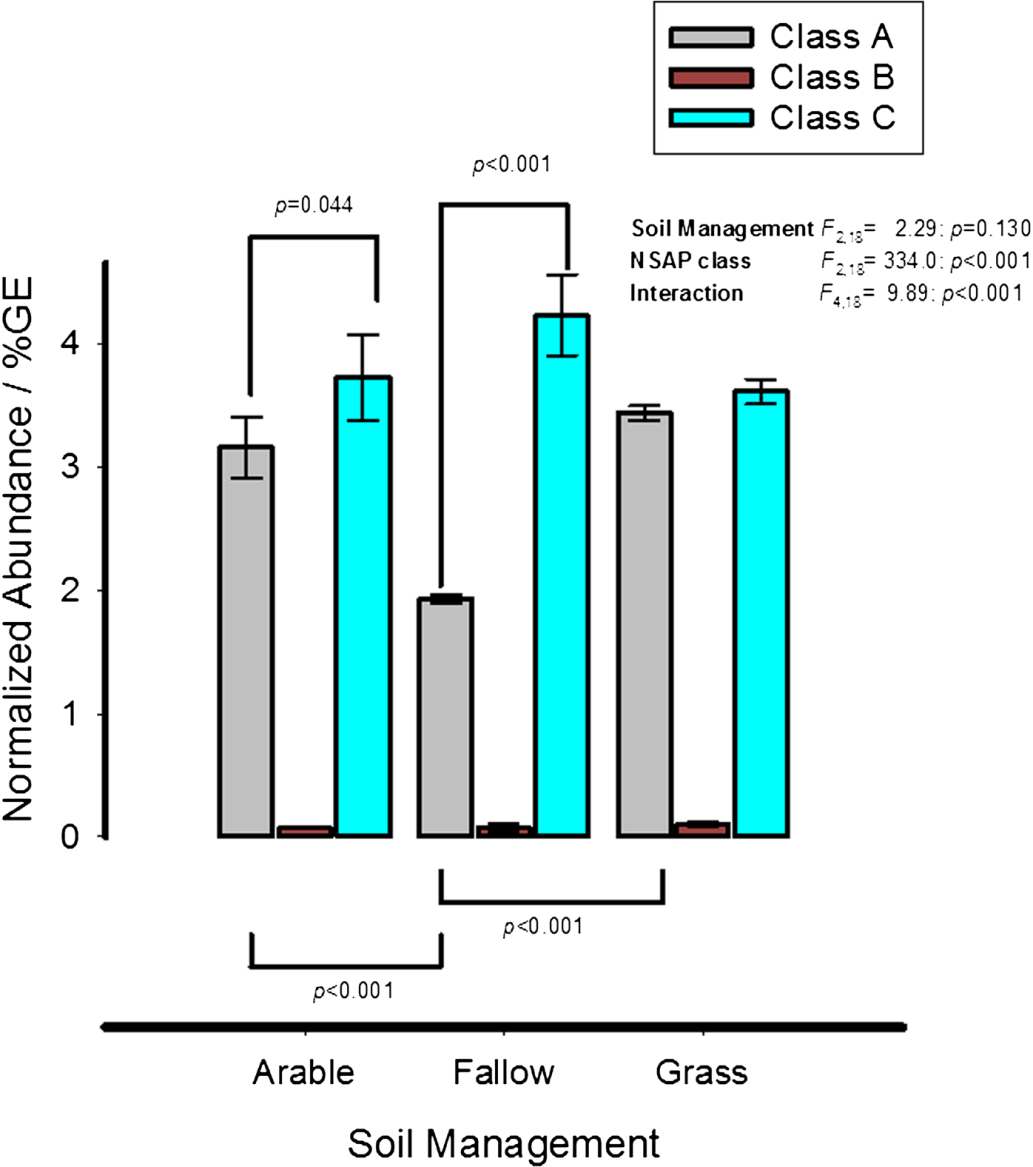
Soil phosphorus speciation determined from solution ^31^P–NMR and normalized abundance of non-specific acid phosphatase gene homologues in shotgun metagenome generated from triplicate datasets for each soil treatment. *A* – estimates of orthophosphate, orthophosphate monoester and dipolyphosphate in alkaline-EDTA soil extracts from the individual grassland, arable and bare fallow plots of the Highfield Ley-Arable experiment. A 50 μg-P L^−1^ methylenediphosphonic acid internal standard was used to estimate the concentration of each species in the soil extracts (see text for details). *B* - Counts of homologous sequences normalized relative to the number of genome-equivalents in each metagenome, normalization details are given in supplementary information. The mean of three replicate plots and standard error are shown for each gene in each treatment. Brackets joining treatment bars show significant Holm-Šídák *post*-ANOVA comparisons

The normalized abundances of the three NSAP classes were determined in triplicate shotgun metagenomes developed from the three soil managements (Fig. 4). As for the previous set of metagenomes, Class B NSAPs were present in extremely low relative abundance (< 0.1%GE). However, classes A and C were present, depending upon the soil management, at between 1.9–4.2%GE. The two classes responded differently to soil management: the proportion of class A NSAPs in bare fallow soil was significantly reduced compared to either arable or grassland soils, in contrast to class C genes where no significant effect of soil management was observed. The result was that although the two classes were present in grassland soil in equal relative abundance, class C genes comprised a significantly greater proportion in arable and particularly, bare fallow soil.

**Fig. 5:**
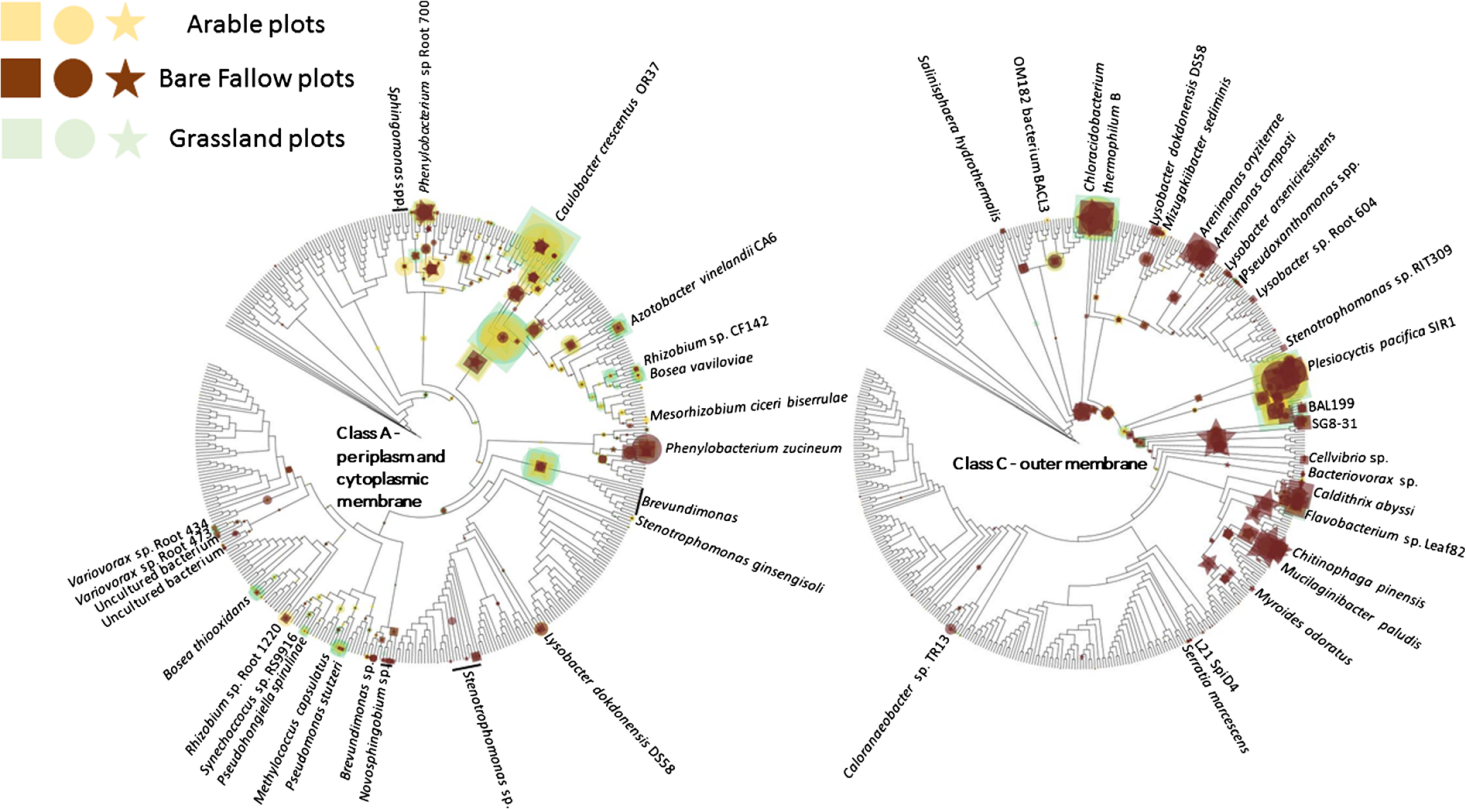
Phylogenetic placement of metagenome sequences generated from contrasting land management plots of the Highfield Ley-Arable experiment showing homology to class A and class C non-specific acid phosphatase reference gene sequences. The placement of reads from each of the nine metagenomes are overlaid for each maximum-likelihood tree and are represented by different symbols. The normalized abundance of each ecotype (accumulation of reads) is represented, normalized to the maximum abundance – homologues of *Caulobacter crescentus* OR37 in grassland in the case of Class A and reads placed at an internal node, again in the grassland in the case of Class C. The size of the symbol representing read placements is proportional to the normalized relative abundance. For clarity, organisms harbouring homologous gene sequences are only identified where they are associated with read placements

Class A NSAP ecotypes in grassland and arable soils were dominated by sequences with high homology to genes of *Caulobacter crescentus* OR37, *Granulibacter bethesdensis*, a placement with reduced homology to a clade including *Pseudomonas*, *Serratia*, and *Desulfovibrio* and a third with reduced homology to a *Brevundimonas* clade (Fig. 5). In all cases, the abundance of these ecotypes was higher in grassland and arable soils than in bare fallow soil. Other ecotypes characteristic of grassland and arable soils but present in lower abundance showed high homology to *Azotobacter*, *Rhizobium*, *Bosea*, *Methylococcus* and *Synechococcus*. Class A ecotypes which were more abundant in the bare fallow soil showed high homology to *Phenylobacterium* and at reduced abundance to *Variovorax*, *Sphingobium*, and *Novosphingobium*.

For class C NSAPs, placement of homologous metagenome reads on the phylogenetic tree indicated a separation between ecotypes present in grassland and arable soils and those dominating bare fallow soils (Fig. 5). Ecotypes associated with the former exhibited high homology to a clade containing NSAPs from the myxobacterium *Plesiocyctis* and *Azospirillum*, *Inquilinus* and *Rhizobium*. A second group of common metagenome sequences showed high homology to *Chloroacidobacterium thermophilum* class C NSAP and a third group exhibited reduced homology to a clade composed of the *alphaproteobacteria Martelella*, *Thioclava*, and BAL199. In contrast, there were a number of metagenome sequence placements which were characteristic of the bare fallow soils: these included ecotypes with high homology to *Arenimonas oryziterrae* and the closely related *A. composti*, *Chlorobium*, *Chitinophaga*, *Mucilagibacter* and placements with reduced homology to *Elizabethkingia*, *Pedobacter*, *Myroides* and *Chryseobacterium*. None of the ecotypes were exclusive to any soil but the abundance of these reads was much greater in bare fallow soil.

#### **Edaphic factors influencing NSAP gene distribution**

**Fig. 6:**
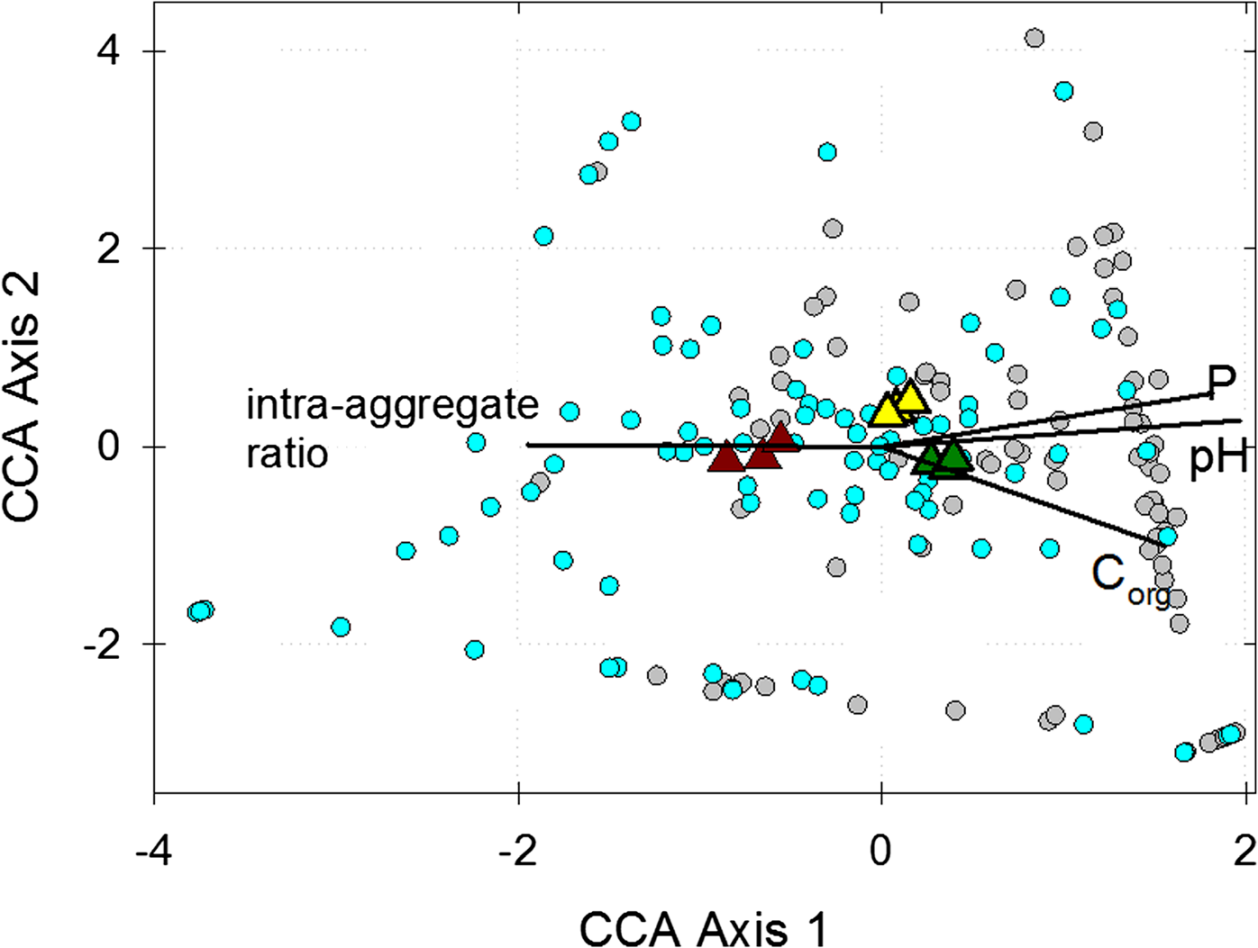
Gene-conditional triplot based upon canonical correspondence analysis of class A and C non-specific acid phosphatase ecotypes from the Highfield Ley-Arable experiment presented in Fig. 5, and edaphic factors. Individual ecotypes are represented as circular data points and distinguished as either class A (grey) or class C (blue) genes. Triangles represent the centroids of individual replicate plots of grassland (green), arable (yellow) and bare fallow (brown) soils with regard to the ordination of relative frequencies across gene ecotypes. Environmental factors - pH, NaOH-EDTA extractable orthophosphate (P), C_org_ and intra-aggregate ratio - are represented as vectors and increase in the direction of the vector: vector length indicates the degree of correlation of each environmental variable with ecotype relative frequencies. Total inertia constrained by the model = 0.2791 (*p* = 0.0029). The eigenvalue for Axis 1 = 0.186 (*p* = 0.0033), representing 66.5% of the variance, and for Axis 2 = 0.054 (*p* = 0.160), representing 19.4% of the variance

To identify soil physical and chemical factors likely to influence gene distribution, responses of Class A and C NSAP ecotypes to soil chemical and structural factors were assessed using CCA. The resulting gene-conditional triplot is shown in Fig. 6 and a clear separation of class A and class C ecotypes on Axis 1 is evident. The median score for class A ecotypes (0.969, 90% confidence interval 0.572 to 1.212) is significantly higher than that for class C ecotypes (−0.354, 90% confidence interval − 0.679 to 0.015) on Axis 1 (Mood test, χ^2^ = 30.48; *p* < 0.0001). The edaphic factors (shown as vectors on the triplot) most strongly associated with Axis 1 are pH and intra-aggregate ratio: increasing axis scores are associated with increasing pH but decreasing intra-aggregate ratio (alternatively, an increase in free C_org_). Extractable orthophosphate was less strongly associated with Axis 1 while C_org_ was only weakly associated.

## **Discussion**

NSAPs are a phylogenetically diverse group of enzymes found predominantly in Gram-negative bacteria. They remain relatively poorly studied and so the significance of NSAPs to the turnover of organic P in the environment is unclear. Searches of the UniprotKB protein sequence database identified a large number of proteins from each of the three NSAP classes. From these, a number of conserved amino acid motifs characteristic of each class have been identified (Fig. 1): although these are not novel we have expanded the number of sequences described previously by Rossolini et al. (1998) and Gandhi and Chandra (2012) to include 1900 non-redundant enzymes across the three classes, with class C being the most numerous. The three classes appear to have distinct sub-cellular localization profiles with enzymes that can be classified from classes A and B being periplasmic and associated with the cytoplasmic membrane while those from class C are associated with the outer membrane and thus able to catalyse the hydrolysis of large organic compounds. This evidence suggests that this class is most responsible for the release of P from soil organic matter among the NSAPs. This conjecture is supported by the fact that class C gene homologues are more abundant than class A homologues in all but one of the eight metagenomes from diverse environments and Highfield soils.

Class B genes are least common in all of the soils or sediments tested here (Fig. 2), perhaps reflecting an apparent association with enteric and pathogenic bacteria. Distinct class A and class C gene ecotypes are associated with marine and terrestrial environments but within those studied here the marine systems Noosa River and Columbia River estuaries appear to be particularly divergent, each dominated by quite different ecotypes, particularly of class C genes. Although NSAPs in general do not appear to be abundant in the bathypelagic marine environment, they are relatively more common in marine rather than terrestrial systems. In comparison, homologues from terrestrial systems appear more diverse, particularly those of class A genes, the dominant ecotype and number of ecotypes varying from hydric peat bog and paddy soils to tallgrass prairie and hot arid desert environments.

The hydric peat bog from the Marcell Experimental Forest is unique among the metagenomes studied here in that class A gene homologues comprise a higher proportion than class C. Previous studies have demonstrated that acid phosphatases (Pfam family PF01451.16) in general are more abundant than alkaline phosphatases (PF00245.15) in this acid (pH 3.5–4.0) oligotrophic soil (Lin et al. 2014). In contrast, Fricke Cemetery prairie (pH = 6.5, Fierer et al. 2013), Uluṟu (pH = 6.8, https://downloads.bioplatforms.com/base/contextual/sample/102.100.100.8160) and Rothamsted Highfield (pH 5.1–6.0, Gregory et al. 2016) soils are less acidic and in each case class C homologues are more abundant than class A. This is also the case with the other four environments (unfortunately, pH is not consistently reported for the datasets used in this study) where the pH of submerged paddy soils is often in the range of 6–7 (Yu 1991) and marine sediments are typically of circumneutral to alkaline pH (Gaillard et al. 1989; Zhu et al. 2006).

Niche separation of class A and C NSAPs is evident from comparison of Highfield soils (Figs. 5 and 6) where three class A ecotypes showing homology to *Caulobacter crescentus*, a *Brevundimonas* clade and a group of reads placed deep on the cladogram, are much more abundant in grassland and arable soils compared to bare fallow soil. Plant roots often generate sites of low pH in soil (Hinsinger 2001) that may act as a niche for class A NSAPs. Support for this comes from zymographic imaging of phosphatase activity associated with the Lupin rhizosphere (Spohn and Kuzyakov 2013) which identifies acid phosphatase activity of indeterminate origin associated closely with roots which is expressed irrespective of P status (cf. *phoC*, a class A NSAP of *Morganella morganii*) and alkaline phosphatase activity associated with the surrounding soil. Besides the apparent association of the dominant class A ecotypes with plants, contrasting management of Highfield soils has other marked effects upon NSAP abundance which again highlights trait differences between class A and class C genes. Unlike class A genes, there is no significant effect of land management upon class C gene abundance (Fig. 4). In fact, the relative abundance of class C genes increases from grassland soil to arable soil and is greatest in bare fallow soil; despite overall microbial abundance being the least in bare fallow soil (Hirsch et al. 2009; Hirsch et al. 2016). It is clear that the outer-membrane associated class C enzymes are less sensitive to the effects of reduced nutrient availability and soil structure associated with bare fallow management. Organic matter in this soil is predominantly intra-aggregate (Hirsch et al. 2009) in contrast to the other two soils: not only is there less P overall and the lowest amounts of orthophosphate monoester, but access to it is likely to be more challenging. A number of class C ecotypes are much more abundant in bare fallow soil than either arable or grassland soils. These include homologues to *Arenimonas* spp., *Chlorobium* spp., and *Pedobacter* spp. clades amongst others. CCA ordination (Fig. 6) presents alternative possible explanations for separation of NSAP classes across the experiment: either class C genes are more characteristic of acidic environments than class A (counter to the consideration of pH above), or class C genes are associated with environments in which organic material is difficult to access because of incorporation in soil aggregates. Although pH and intra-aggregate ratio are confounded in the Highfield plots, two independent lines of evidence suggest the latter provides a more satisfactory explanation to the distribution of the two genes. The first is that reported in vitro pH optima for PhoN, a class A NSAP (pH 5.5), and CppA, a class C NSAP (pH 6.0) are similar (Reilly et al. 2009; Makde et al. 2006), although the environmental pH optima of the range of ecotypes described in this work are difficult to assess (see for example Turner 2010). The second line of evidence is that Class C proteins are exoenzymes associated with the surface of the outer membrane while Class A proteins for which we were able to predict a subcellular location are either periplasmic or associated with the cytoplasmic membrane. This suggests that structure-related phenomena, both in terms of occlusion of organic material within aggregates and the presentation of enzymes on the outer membrane surface interact, with the result that class C genes are more dominant in bare fallow soil. These data suggest that under conditions of poor P-availability and access, free-living bacteria encoding outer membrane-associated NSAPs have an advantage over the more predominantly plant-associating organisms harbouring intracellular NSAPs. The ratio between the two enzymes appears to be directly influenced by soil physicochemistry, particularly soil structure.

## Abbreviations

CCA: Constrained canonical analysis
C_org_: Organic carbon
DNA: Deoxyribose nucleic acid
*E*: Expect value
EC: Enzyme commission number
EDTA: Ethylenediaminetetraacetic acid
FAO: Food and Agriculture Organization of the United Nations
NMR: Nuclear magnetic resonance
ML: Maximum likelihood
N: Nitrogen
NSAP: Non-Specific Acid Phosphatases
P: Phosphorus
pHMM: Profile hidden Markov model
%GE: Percentage of genome equivalents
